# Re-work Program in Japan—Overview and Outcome of the Program

**DOI:** 10.3389/fpsyt.2020.616223

**Published:** 2021-01-18

**Authors:** Yoko Ohki, Yoshio Igarashi, Keita Yamauchi

**Affiliations:** ^1^Keio Research Institute at SFC, Fujisawa, Japan; ^2^Tokyo Institute of Rework for Depression, Tokyo, Japan; ^3^Medical Care Ohtemachi Clinic, Tokyo, Japan; ^4^Japanese Association of Rework for Depression, Tokyo, Japan; ^5^Graduate School of Health Management, Keio University, Tokyo, Japan

**Keywords:** return to work, sick leave, mood disorder, treatment outcome, occupational health

## Abstract

**Background:** The objective of this study was to examine the effect of the Japanese re-work program (RP) to aid in recurrent sick leave prevention.

**Methods:** A multicenter retrospective cohort research was conducted for workers who returned to work (RTW) after sick leave due to mood disorder. Work continuation for subjects who RTW after RP participation and treatment as usual (TAU) and subjects who received TAU only were compared. The Kaplan–Meier method and Cox proportional hazard models were utilized. Additionally, propensity score matching was conducted to control for possible confounds.

**Results:** Log-rank test of overall cohort (*n* = 323) showed that work continuation of RP + TAU subjects was significantly better compared to that of TAU-only subjects (*p* = 0.001). Multivariate analysis found a hazard rate of recurrent sick leave for TAU-only subjects of 2.121 (*p* = 0.001, 95% CI: 1.360–3.309). Additionally, the propensity score-matched cohort (*n* = 100) had similar differences (*p* = 0.008), with a hazard ratio of recurrent sick leave of 2.871 (*p* = 0.009, 95% CI: 1.302–6.331) for TAU-only subjects.

**Limitations:** Only workers who RTW after sick leave were targeted, and no examination was made considering cases who dropped out from RP or TAU. Moreover, the sample was a non-randomized controlled trial, with propensity score matching performed. However, there was an inability to retrieve and adjust for working environment background factors after RTW.

**Conclusions:** Work continuation of subjects with RP was observed to be significantly better, suggesting that the RP was effective for recurrent sick leave prevention.

## Introduction

Mental health problems, including depression, are a global concern. Mental health issues of workers are also an important topic in the occupational field, with studies examining the expenses for illness in developed countries reporting significant economic loss due to depression (1, 2). Similarly, mental health problems of workers are also one of the most urgent issues in Japan, with the “stress check program in the workplace” enforced according to the Industrial Safety and Health Act in 2015 (3). This is to mandate implementation of a stress check for all workers once a year in any corporation with 50 or more employees, which marks a start in efforts to reduce the risk of individual workers developing mental health problems, analyzation of examination results collectively, and a connection of results to improvements in work environment (4).

In recent years, various studies have been conducted in many countries on the effect of support for reinstatement (i.e., return to work, RTW) for workers on sick leave due to mental disorders, including depression (5). Facilities for interventions, content, and the executors of interventions vary, as seen in work-directed interventions (6–10) and clinical interventions (11–14). In general, however, the common objective of these programs is to reduce the sick leave period, and the primary outcome measure is often the number of days to RTW (11, 15–17).

RTW support is also conducted in Japan. However, the support conducted therein is uniquely distinct from that in other countries. Support is conducted under the name of the re-work program (RP). In what follows, the characteristics of Japan's RP are enumerated.

1. Main targets for the RP are those who are repeating sick leave, or who have difficulty in RTW, such as those who are on long-term sick leave, with the ultimate goal of preventing recurrent sick leave.
2. The costs are covered by the public health insurance, with the obligation for patients ranging from 0 to 30%.
3. The sites for implementation are medical institutions, and the program is implemented in parallel and in addition to “treatment as usual” (TAU), including regular medical examinations and drug treatment.
4. The RP is implemented by the psychiatry daycare division, in a cohort, and by the medical staff of multiple professions, including registered nurses, certified clinical psychologists, psychiatric social workers, and occupational therapists implementing the RP under the direction of psychiatrists.

Note that the implementation requires a period of ~6–8 months, depending on the degree of recovery for patients.

The year 1997 witnessed the first implementation of RP in Japan, by Akiyama (18) at the NTT Medical Center, Tokyo (19). Since then, medical institutions that implement the RP have rapidly increased from 2010, with the number increasing to more than 200 as of today. The increasing popularity of this program is also evidenced by the geographical distribution, as at least one medical institution that implements RP exists in 46 out of all 47 prefectures in Japan. However, verification in the outcome has been delayed, due to a rapid increase in number of participants in recent years, and no examination of the scientific effects has been conducted thus far. Therefore, the current study examines the effect of prevention of recurrent sick leave, which is the goal of RP, by comparing work continuation after RTW for subjects who participated in the RP, in addition to TAU, and subjects who received TAU only.

## Methods

### Study Design

This study was conducted as a multicenter retrospective cohort research. A randomized controlled trial is desirable when examining the effect of treatment. However, because the RP has already been covered by public health insurance and some level of effect is already presumed, it is difficult to recommend to patients who were going to visit an RP-providing medical facility a treatment in terms of TAU only by random sampling. Furthermore, because the RP was to be implemented in addition to TAU, blinding was impossible, and it was difficult to have the control group maintain their motivation to participate in the study for long periods. To make matters worse, because the end of an intervention corresponds to the time of RTW, it was impossible to leave the control group as the waiting list control. Thus, a retrospective cohort study was employed.

This study was conducted with the approval of the ethical review committee of the Depression Rework Research Association (H24-03). As this study is retrospective, the data were obtained from re-work facilities. To prevent from identifying an individual at the time of the data acceptance from the facility, the different number from a medical record and the employee number was used. Written informed consent from the patients to participate in this study was not required in accordance with the national legislation and the institutional requirements.

### Setting and Participants

Enrolled period was 4 years, from 2007 to 2011, with follow-up until 2012. The questionnaire survey was sent to the RP implementation staff of medical institutions for subjects with TAU + RP and to the company's health control office staff for subjects with only TAU.

The following two criteria should be met for inclusion. First, the participant must have been a worker who RTW after taking sick leaves due to mental illness either on at least two occasions or for 6 months or longer, even for first-time cases. Second, the participant must continually undergo a medical examination by psychiatrists on a regular basis before RTW and was diagnosed according to the criteria of the ICD-10 (20) with a mood disorder.

### Interventions

#### TAU

TAU included only regular outpatient practices and medication by psychiatrists, and subjects who utilized the RP and other RTW support services were excluded. If subjects of TAU were assembled from medical institutions that offered the RP, confounding by indication could occur, as the attending doctor could advise the patient to use RP. Data sources in health control offices in 2 prefectures and 25 companies were used to collect target data from a larger number of facilities. The data sources were organized by industrial physicians and industrial health staff based on the medical examination information provision form created by the attending psychiatrist of the subject as well as the subject's self-report.

RTW was determined by the final judgment of the company at which the subject was employed, based on the diagnosed permission of RTW by the attending psychiatrist.

#### TAU in Conjunction With RP

Medical records in institutions that offered the RP were used. Facilities that met the general standards of the Japanese RP were selected as the medical institutions that would run the RP. In concrete terms, six medical institutions in five prefectures were selected, in light of regional characteristics, out of medical institutions with four or more years of experience, based on fulfillment of conditions that the institution runs the RP 5 days or more in a week and the programs they offer are varied. These six medical institutions were Sapporo Ekimae Clinic (Hokkaido), Shinagawa Ekimae Mental Clinic (Tokyo), Himorogi Psychiatric Institute (Tokyo), Kyoto Ekimae Mental Clinic (Kyoto), Sakura Clinic (Osaka), and Kawano Clinic (Fukuoka). All are psychiatric clinics, and two facilities are satellite clinics of mental health hospitals. The RP experience of the six medical institutions was 5.7 years (SD 1.4) on average, and the RP was implemented 5.3 days (SD 0.5) a week on average, which primarily involved psychiatry daycare (6 h/day) for which medical treatment fees were incurred. The mean number of patients taking the program was 37.5 (SD 19.4).

In Japan, the RP was implemented based on a standardized program (21), which is roughly classified into five types: (1) individual program, in which the program is implemented with the goal of reviewing and improving concentration, work capability, and practical ability in the course of doing sedentary work, rather than the primary goal of interacting with other participants; (2) specific psychology program, which involves cognitive behavioral therapy, social skills training, interpersonal psychotherapy, group counseling, psychodrama, etc. The program contents of RP are decided in respective facilities, but a program of the medical psychology education mainly on mood disorders is performed by the educational program. About the psychology program, in majority of cases, group CBT program is implemented, which designed for mood disorders. In addition, as the group therapy, the program is conducted in the environment where simulated workplace. (3) educational program, which is implemented in a lecture style with the main goal of understanding the disease and self-understanding of its symptoms (self-monitoring and control); (4) group program, which is carried out in groups based on a clear intent, with the primary objectives of work cooperation, role allocation, and social skill improvement; and (5) other programs, which are programs that do not fall under any one of the previous four categories, such as exercise and personal interviews.

An attending psychiatrist determined the end of the RP, that is, the patient's readiness to RTW, based on assessment provided by staff who are involved in the RP program in various job categories, including registered nurses, certified clinical psychologists, psychiatric social workers, and occupational therapist, as well as on regular medical examinations that were concurrently performed. Then, the company made the final judgment of RTW, based on the psychiatrist's determination.

### Outcome Measure

The number of continuous working days served as the index for the recurrent sick leave prevention effect of the RP. If the subject had repetitive sick leave and RTW during the probation period, the period from the referential RTW day to the day prior to the first day of sick leave was taken as the number of continuous working days.

In this study, RTW and “recurrent sick leave” are defined as follows. RTW refers to the state of starting to work in practice, after the final judgment of the company the subject works for, regardless of whether the subject is a full-time or part-time employee. “Recurrent sick leave” refers to the state of taking leave continually for a certain period of time due to mental illness, after RTW and the issuance of a doctor's certificate by the attending psychiatrist.

### Variables

As the basic variables, the RTW day was taken as the baseline, and sex, age, primary diagnosis, number of sick leaves, total sick leave days, type of business, and company size were investigated. As a diagnosis name, we received answers based on the criteria of ICD-10. Of which, we have defined Bipolar affective disorder as Bipolar disorder, and categorized Depressive episode, Recurrent depressive disorder, and Persistent mood [affective] disorders as Unipolar disorder. As for type of business, because half of the subjects who took only TAU belonged to the information technology industry, subjects were categorized broadly as either belonging to the information technology industry or to other industries. According to the 2012 Survey on State of Employees' Health of the Ministry of Health, Labor and Welfare in Japan (22), their findings have shown that out of the 17 industries, number of workers who have retired or been on the sick leave for more than one month, the IT industry is by far the largest. There is no doubt that there are various types of occupations in IT industry as well as other industries. However, especially IT industry is conspicuous in regard to the large number of sick leave and retirement due to mental health problems. This is the reason why we have chosen this variable. Furthermore, company size category was divided into < 1,000 employees and 1,000 employees or greater. In Japan, the Industrial Safety and Health Act mandates employment of an industrial doctor if a company regularly employs 50 or more individuals regularly or the employment of a full-time industrial doctor if a company regularly employs 1,000 or more individuals. Therefore, not only does the unit of 1,000 employees represent the company size, it also serves as a rough indication of the company's industrial health management organization.

### Sample Size

Based on data obtained through a pilot study (23) conducted for the purpose of this study, the hazard ratio for TAU recurrent sick leave was set to 2.9, detection capability to 90%, level of significance to 5%, and assignment ratio to 1:1, and the necessary sample counts were estimated to be 84 total subjects (42 subjects per group).

### Statistical Analysis

Analysis was carried out in the following two levels.

#### Overall Cohort Analysis

An overall cohort analysis was initially performed on all subjects who met the inclusion criteria. To compare the baseline, a χ^2^ test was used for the qualitative variables, and a *t*-test or Mann–Whitney *U*-test was used for the quantitative variables.

To examine the effect of the RP in the prevention of recurrent sick leave, the number of continuous working days was compared between the group with TAU + RP and the group with only TAU using the Kaplan–Meier method. The RTW day was set as the starting point of the calculation, and recurrent sick leave, loss of a job, and suicide due to mental illness were set as event cases. Censored cases refer to termination of treatment due to recovery during continued employment or recurrent leave or due to loss of a job for reasons other than mental illness. In addition, factors relevant to continuous employment after RTW were examined. Two groups were combined into one, the RP participation record was added as one of the basic attributes, and a univariate or multivariate Cox proportional hazard model was used for examination. In so doing, ages were divided and categorized into groups for every 10 years.

#### Propensity Score-Matched Cohort Analysis

The current study utilized a non-randomized controlled trial, and because there might be a difference in the attributes between TAU + RP and TAU only, which could be seen as a confounding variable through indication in RP participation, propensity score matching (24, 25) was performed.

Presence or absence of RP utilization was viewed as a dependent variable, and a logistic regression analysis, which is adjusted by interaction variables of number of sick leaves and profession, was performed on all of the aforementioned survey items to calculate the conditional probability (i.e., propensity score) for utilization of the RP. Based on the propensity score calculated, subjects whose probability marked the lowest difference from TAU were matched (i.e., one-to-one pair matching). The balance between covariates before and after the matching was observed in the standardized difference (26, 27).

The effect of the RP on prevention of recurrent sick leave was observed according to the same procedure as that of the overall cohort for subjects extracted through propensity score matching. For the statistical analysis, SPSS 22.0 for Mac was used, and 5% for both poles were deemed significant.

## Results

### Study Sample

A total of 323 subjects (TAU only *n* = 133, RP + TAU *n* = 190) were incorporated into the overall cohort. Subjects exposed to TAU only were treated in 102 medical institutions. The facility types were psychiatric clinics for all six facilities that offered TAU + RP and 90 psychiatric clinics, 8 mental hospitals, 2 university hospitals, and 2 general hospitals for subjects with TAU only. Two subjects received treatment from unknown medical institutions. Note that because the Japanese medical system allows free access and free choice to any medical institution, the patient can freely choose which medical institution to visit. Therefore, regardless of facility type, a fundamental difference in outpatient function is not produced.

**Figure 1:**
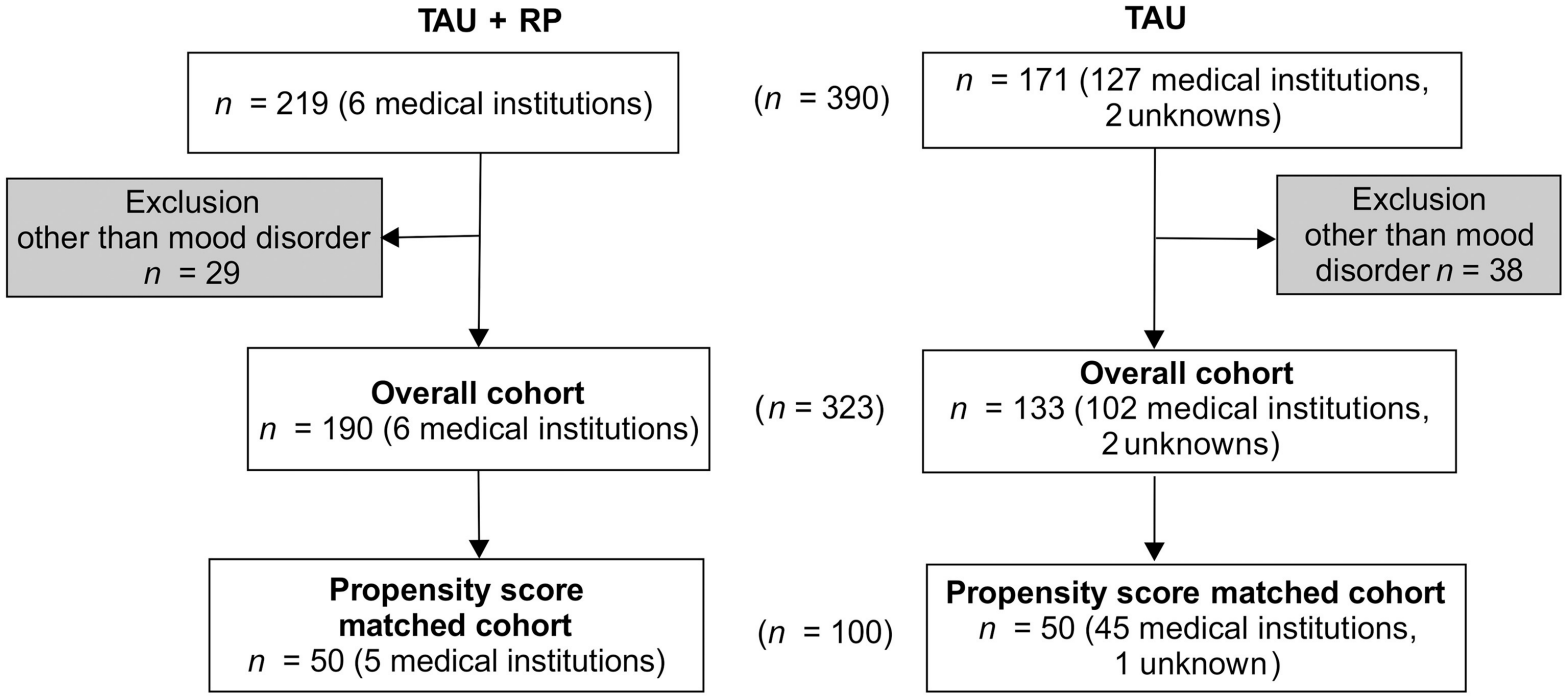
Flow Diagram for Subjects RP = Re-work program; TAU = Treatment as usual.

A total of 100 subjects (TAU only *n* = 50, RP + TAU *n* = 50) were extracted as the propensity score-matched cohort. The range of calculated trend scores was 0.02–0.97 for TAU only and 0.02–1.00 for TAU + RP. Subjects with TAU + RP were exposed to the RP in five medical institutions, while subjects exposed to TAU only received treatment in 45 medical institutions. The facility type was psychiatric clinic for all subjects with TAU + RP and 37 psychiatric clinics, 5 mental hospitals, 2 university hospitals, and 1 general hospital for subjects with TAU only. One subject received treatment from an unknown medical institution (Figure 1).

### Overall Cohort Analysis

The mean follow-up period for the overall cohort was 998.2 days, with a median value of 1,003.0 and SD of 416.5. Comparison of attributes between the two groups yielded significant differences in every survey item other than company size. As for the history of sick leave, sick leave tended to recur, previous total sick leave days tended to be longer, and the number of subjects who experienced difficulty in RTW tended to be higher for subjects with TAU + RP. Furthermore, concerning the mood disorder, the rate of subjects with bipolar disorder also tended to be higher for subjects with TAU + RP (Table 1).

**Table 1:** Attributes of subjects.

	**Overall cohort (*****n*** **=** **323)**						**Propensity score-matched cohort (*****n*** **=** **100)**					
	**RP** **+** **TAU** ** (*****n*** **=** **190)**		**TAU** ** (*****n*** **=** **133)**		***p*-value**	**Standardized difference**	**RP** **+** **TAU** ** (*****n*** **=** **50)**		**TAU** ** (*****n*** **=** **50)**		***p*-value**	**Standardized difference**
	***n*/mean**	**(%/SD)**	***n*/mean**	**(%/SD)**			***n*/mean**	**(%/SD)**	***n*/mean**	**(%/SD)**		
Age	40.6	(7.8)	34.9	(6.6)	*p* ≦ 0.001^***^	0.7776	37.1	(6.9)	37.7	(7.5)	0.688	−0.0833
Sex (male)	164	(86.3)	97	(72.9)	0.003^**^	0.3393	41	(82.0)	41	(82.0)	1.000	0.0000
Diagnosis												
Unipolar disorder	149	(78.4)	120	(90.2)	0.005^**^	0.3152	41	(82.0)	42	(84.0)	0.790	0.0533
Bipolar disorder	41	(21.6)	13	(9.8)			9	(18.0)	8	(16.0)		
Number of sick leaves	2.1	(1.4)	1.6	(1.0)	*p* ≦ 0.001^***^	0.3997	1.8	(0.9)	1.7	(1.1)	0.330	0.0995
Total sick leave days	566.8	(402.9)	365.8	(214.5)	*p* ≦ 0.001^***^	0.5440	443.4	(228.2)	456.4	(289.4)	0.746	−0.0499
Type of business												
Information technology	33	(17.4)	81	(60.9)	*p* ≦ 0.001^***^	1.0031	29	(58.0)	28	(56.0)	0.840	0.0405
Other than information technology	157	(82.6)	52	(39.1)			21	(42.0)	22	(44.0)		
Company size												
<1,000 employees	45	(23.7)	42	(31.6)	0.115	0.1759	17	(34.0)	17	(34.0)	1.000	0.0000
1,000 or more employees	145	(76.3)	91	(68.4)			33	(66.0)	33	(66.0)		

*RP, Re-work program; TAU, Treatment as usual*.

** *p <0.01*;

*** *p ≦ 0.001*.

**Figure 2:**
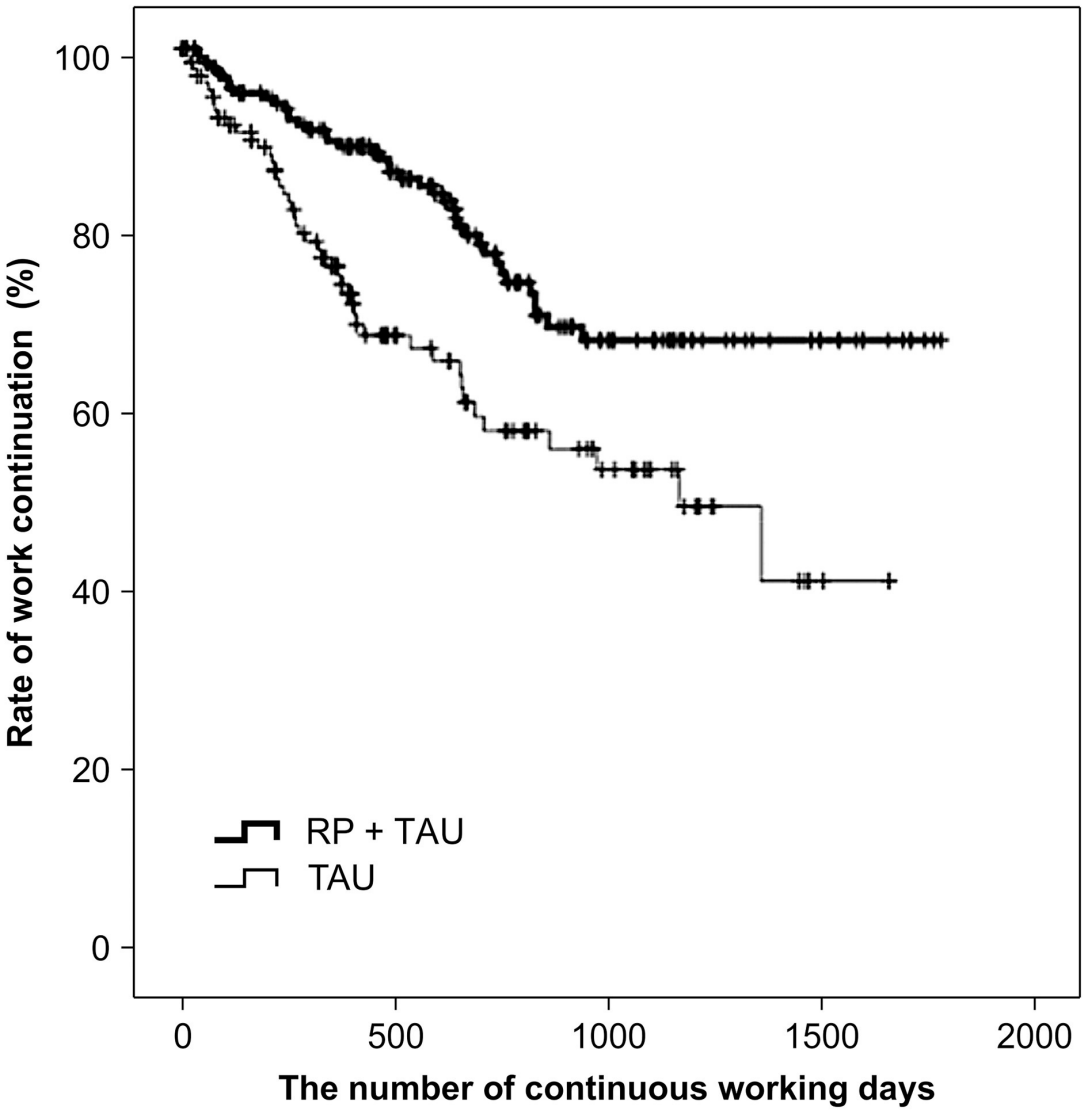
Comparison of work continuation after RTW in overall cohort (*n* = 323) RP = Re-work program; TAU = Treatment as usual: RTW = Return to work.

A comparison of the number of continued working days after RTW, using a log-rank test, showed a significant difference between TAU + RP and TAU only (*p* = 0.001), with the work continuation for TAU + RP shown to be better (Figure 2). Second, factors relevant to work continuation after RTW were examined using Cox proportional hazard models. Age, RP utilization, and type of business showed a significant difference, after the univariate analysis was adjusted along with sex to perform the multivariate analysis (i.e., stepwise forward selection). As a result, RP utilization was identified as a significant, relevant factor. The hazard ratio of recurrent sick leave for subjects with TAU only was 2.121 (*p* = 0.001, 95% CI: 1.360–3.309), and work continuation was shown to be significantly better for TAU + RP (Table 2).

**Table 2:** Study on the recurrent sick leave factors after RTW observed in overall cohort (*n* = 323).

		**Univariate Cox regression**			**Multivariate Cox regression**		
		***p***	**Hazard ratio**	**95% CI**	***p***	**Hazard ratio**	**95% CI**
Age (years old)	20–29 [ref]	0.040^*^	1		0.073	1	
	30–39	0.010^**^	0.479	0.273–0.841	0.012^*^	0.485	0.276–0.852
	40–49	0.010^*^	0.440	0.236–0.822	0.064	0.547	0.289–1.036
	50–59	0.236	0.626	0.288–1.358	0.669	0.840	0.379–1.863
Sex	Male [ref]/female	0.915	1.029	0.613–1.727			
Diagnosis	Unipolar[ref]/bipolar	0.548	0.843	0.483–1.471			
The number of sick leaves		0.389	1.069	0.918–1.246			
Total sick leave period (month)		0.201	1.011	0.994–1.028			
RP utilization	RP + TAU [ref]/TAU	0.001^**^	2.067	1.358–3.146	0.001^**^	2.121	1.360–3.309
Type of business	Information technology [ref]/others	0.009^**^	0.574	0.377–0.872			
Company size	<1,000 employees [ref]/1,000 or more employees	0.665	1.117	0.678–1.839			

*RTW, Return to work; RP, Re-work program; TAU, Treatment as usual; 95% CI, 95% confidence interval*.

* *p < 0.05*;

** *p <0.01*.

### Propensity Score-Matched Cohort Analysis

The mean follow-up period for the propensity score-matched cohort was 980.8 days, with a median value of 1,030.5 days and SD of 409.9. Observation of the balance of the covariance between the two groups before and after matching, through examination of the standardized difference, found that covariance values after matching fell below 0.1, which is generally considered to be well-balanced. In addition, no significant difference was found in all survey items in attribute comparisons (Table 1).

**Figure 3:**
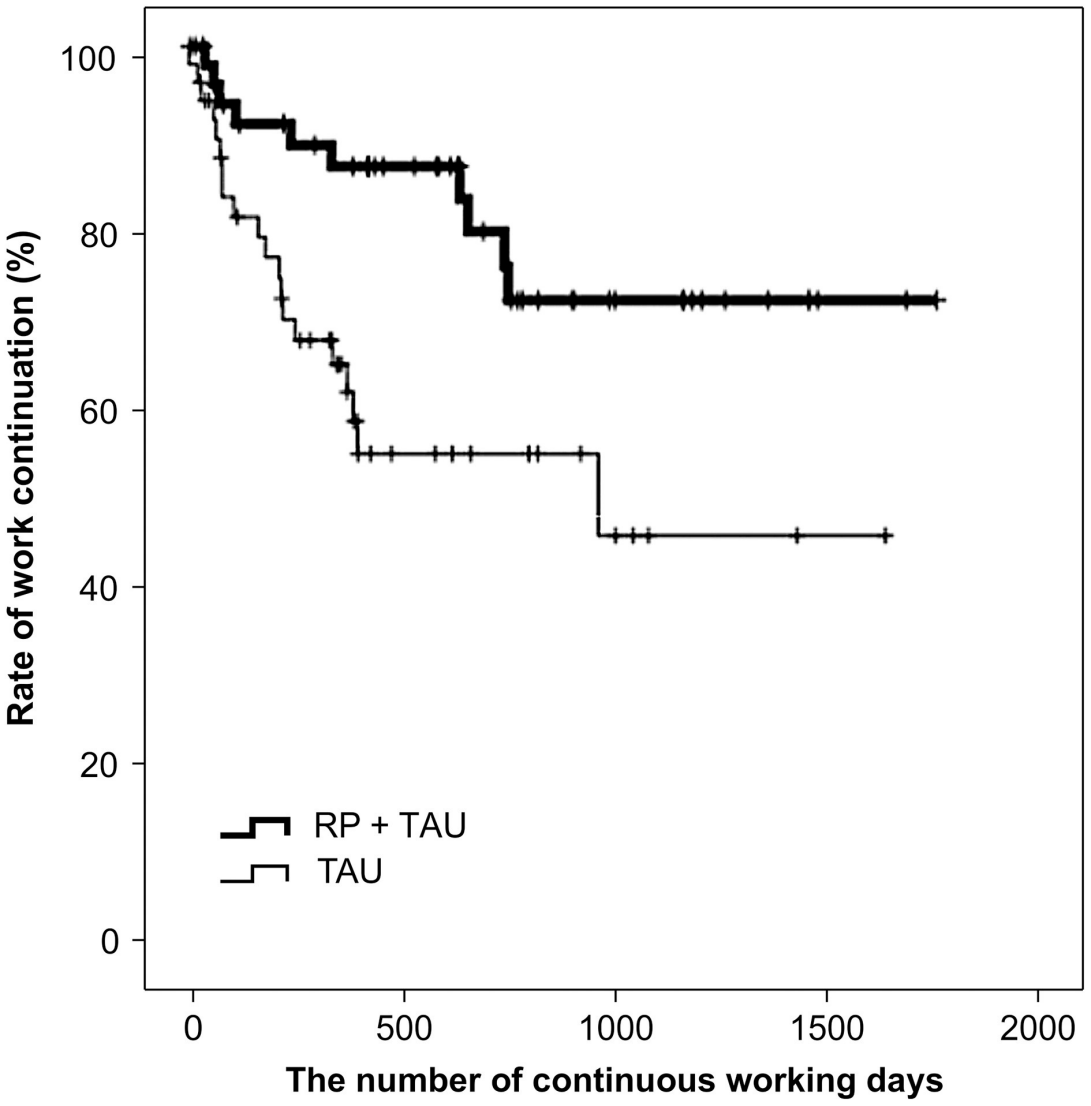
Comparison of work continuation after RTW in propensity score matched cohort (*n* = 100) RP = Re-work program; TAU = Treatment as usual: RTW = Return to work.

A comparison of the number of continued working days after RTW using the log-rank test showed a significant difference between TAU + RP and TAU only (*p* = 0.008), with the work continuation for TAU + RP shown to be better (Figure 3). In addition, factors relevant to work continuation after RTW were examined using a Cox proportional hazard model in an identical manner to the overall cohort analysis. Age and RP utilization showed a significant difference after the univariate analysis was adjusted along with sex to perform the multivariate analysis (i.e., stepwise forward selection). As a result, RP utilization was identified as a significant, relevant factor. The hazard ratio of recurrent sick leave for subjects exposed to TAU only was 2.871 (*p* = 0.009, 95% CI: 1.302–6.331), with work continuation shown to be significantly better for the TAU + RP group (Table 3).

**Table 3:** Study on recurrent sick leave factors observed in propensity score-matched cohort (*n* = 100).

		**Univariate Cox regression**			**Multivariate Cox regression**		
		***p***	**Hazard ratio**	**95% CI**	***p***	**Hazard ratio**	**95% CI**
Age	20–29 [ref]	0.009^**^	1		0.006^**^	1	
	30–39	0.024^*^	0.379	0.163–0.883	0.007^**^	0.302	0.127–0.720
	40–49	0.001^**^	0.137	0.042–0.449	0.001^**^	0.131	0.040–0.428
	50–59	0.233	0.450	0.121–1.671	0.084	0.307	0.080–1.173
Sex	Male [ref]/female	0.283	0.561	0.195–1.611			
Diagnosis	Unipolar[ref]/bipolar	0.644	0.797	0.304–2.089			
The number of sick leaves		0.775	0.948	0.660–1.364			
Total sick leave period (month)		0.663	0.990	0.947–1.036			
RP utilization	RP + TAU [ref]/TAU	0.011	2.712	1.259–5.842	0.009^**^	2.871	1.302–6.331
Type of business	Information technology [ref]/others	0.477	0.764	0.363–1.606			
Company size	<1,000 employees [ref]/1,000 or more employees	0.793	0.903	0.422–1.934			

*RP, Re-work program; TAU, Treatment as usual; 95% CI, 95% confidence interval*.

* *p < 0.05*;

** *p < 0.01*.

## Discussion

The effect of prevention of recurrent sick leave must be examined according to the temporal order. First, the appropriateness of time of RTW will be considered. In Japan, the final judgement for RTW is made by the company the subject works for, based on the diagnosed permission of RTW by the attending doctor. Therefore, the company does not proceed to a final discussion for RTW unless there is diagnosed permission by the attending doctor. The Ministry of Health, Labor and Welfare has released an RTW support manual for companies to epitomize this series of steps (28). The manual warns that the diagnosed permission by the attending doctor often determines the possibility of RTW according to the level of recovery from symptoms, which does not immediately sanction a judgement for a work performance capability recovery required at the workplace. Thus, the judgement for RTW requires observation of recovery in aspects of both clinical symptoms and social functions. The RP aims at recovery of social functions, such as the profession function. Because it is implemented in a quasi-professional environment in a group, recovery from clinical symptoms as well as social functions can be observed, which allows doctors to make a more realistic judgment about RTW. Concrete information is missing as to how attending doctors for subjects receiving TAU only in this study came to give diagnosed permission for RTW; therefore, a speculative possible reason for this may be that sufficient observation of the recovery of social functions did not occur. In other words, it is possible that the difference in recovery level of social functions between subjects with TAU only and subjects with TAU + RP at the time of RTW gave rise to the differences in work continuation exhibited in the current study, due to a discrepancy in judgements of appropriate RTW timing.

Second, work continuation after RTW will be considered. In the overall cohort, subjects with TAU + RP had a significantly poor history of sick leave and had a significantly higher number of subjects with RTW difficulty. Nonetheless, work continuation was significantly better for subjects with TAU + RP. As the next step, propensity score matching was performed to adjust attributes in both groups, and the difference increased even more. In principle, the RP was a program for recovering social functions with the ultimate goal of preventing recurrent sick leave, so the outcome can be said to be the result of proper functioning of the program. Moreover, the working environment to which a subject returned and the job role individually varied for each subject, and we could not identify them prior to examining the effect of sick leave prevention. The RP is rehabilitation in groups, and there are limitations in how far individual care can be provided. However, we constructed a quasi-workplace environment that utilizes multiplicity within a group, assuming various workplaces, and aimed to recover social functions within that context. It is possible that utilization of the RP allows subjects to easily adjust the workload they encounter after RTW by themselves, which led to a prevention of recurrent sick leave.

Furthermore, as the disease attribute of subjects, the rate of bipolar disorder was significantly higher in subjects exposed to TAU + RP. Because bipolar disorder has a higher frequency of recurrence and has higher frequency of the recurrent disease phase, it reportedly exhibits difficulty in professional settings (29). The RP is implemented for subjects who have repeated sick leave or who have taken protracted leave, which is considered to account for the higher rate of bipolar disorder in subjects who utilized the RP. Bipolar disorder tends to be neglected by doctors compared to other psychiatric disorders (30). There were cases, however, that through long-term observation by staff of various professions through the course of the program revealed that subjects suffered from bipolar II disorder. Connecting the program to implementation of proper drug treatment may have enhanced long-term treatment effects.

In the latest Cochrane review (31), recent 23 studies from Europe, North America and Australia were added. In comparison to other studies, ours are unique to following points. First of all, our study is unique to the extent that we have studied group therapy using the psychological technique whereas there is not such an intervention in 23 studies quoted in this review article. Secondly, a purpose of the intervention is different from 23 studies. Many other studies are intended to decrease sick leave period. However as for this study, we have set a target onto patients who repeat the sick leave or who prolong the sick leave period, and the purpose of our intervention is to prevent recurrent sick leave. Therefore, outcome measure of this study is working continuation days after RTW. Third, the follow-up period of our study is longer than others by large. The average days of our follow-up period was 998.2 days in order to perform prognostic confirmation. Whereas there were only a few studies that follow-up period last more than a year, and most of reviews are about studies with follow-up only last short and medium-term. In other words, the intervention method, purpose and observation period in this study are different from those studies. This difference might be associated with the difference in working style among Europe, America and Japan.

### Limitations

Only workers who RTW after sick leave were targeted, and no examination was made considering cases who dropped out from RP or TAU. There are also cases where dropouts do not return to work, and an examination that takes these cases into account will be necessary. However, although the number of continued working days was taken as the index of the effect, many different cases can be presumed, even among cases where subjects had to take a recurrent leave of absence, such as a case of returning to work right after a short period of leave and continuing to work thereafter or a case of taking an extended leave. In cases of recurrent sick leave then, it is necessary to track the subsequent working status and to examine the working rate over a certain period.

Because this study is based on a non-randomized controlled trial, matching using covariate adjustment in terms of a propensity score was performed. However, there is a limitation of not being able to retrieve and adjust background factors, such as working environment, etc.

Also, medication treatment has not been concerned in this study because such treatment is ordinary implemented in both groups. While intervention and follow-up period, it is important to observe the change and effect of medication treatment, we admit.

In Japan, it is commonly known that many system engineers who work long and irregular working hours tend to suffer from mental illness. In our study, a majority of our participants who received only TAU, have belonged to IT firms. This is the reason why we have adopted such categorization. However, it is true that all different sorts of jobs and roles (administration, sales and other technological roles) exists within IT industry just like as the others. From this perspective, it would be the ideal if questions about their occupation were included. We admit that this is the limitation of this study.

### Future Research

The working environment varies largely depending on region. For this reason, multiple facilities were selected as targets of the medical institutions that provide the RP, and a model was also selected that included as many psychiatric medical institutions as possible for subjects who were exposed to TAU only, to address generalizability of results. However, after creation of the protocol for this study, the number of medical institutions that provide the RP rapidly increased from ~100 to more than 200 over 4 years. Due to such circumstances, it is necessary to examine the quality of the program once again and then examine the effect of increasing RP facilities further.

In this study, the number of continued working days after RTW was treated as an outcome index in the discussion of the effect of RP on the prevention of recurrent sick leave. However, social impact varies greatly, even for subjects who continue to work, depending on whether the subject delivers his or her best performance, or the subject manages to continue to work while delivering poor performance, as clinical symptoms worsen for the subject, to the point of considering recurrent sick leave. According to a study on expenses for diseases in Japan, the total expenses for depression are estimated to be two trillion yen, morbidity expenses due to loss of work productivity, such as absenteeism or working while sick (i.e., presenteeism), cover 45% of the total (32). We want to include as part of our challenges, henceforth, an examination of medico-social effects based on the societal perspective, such as evaluation of presenteeism, in addition to effects from the clinical perspective.

## Conclusion

This study examines the effect of the RP on the prevention of recurrent sick leave, by comparing work continuation status after RTW between workers exposed to TAU only and workers exposed to TAU + RP. Examination was conducted in an overall cohort and propensity score-matched cohort. As a result, in both cases, individuals exposed to RP were observed to exhibit significantly better work continuation status, which suggests that the RP is effective for preventing recurrent sick leave.

## Data Availability Statement

The original contributions presented in the study are included in the article/supplementary materials, further inquiries can be directed to the corresponding author/s.

## Ethics Statement

This study was conducted with the approval of the ethical review committee of the Depression Rework Research Association (H24-03). As this study is retrospective study, the data were obtained from rework facilities. For prevention from identifying an individual at the time of the data acceptance from the facility, the different number from a medical record and the employee number was used. Written informed consent from the patients was not required to participate in this study in accordance with the national legislation and the institutional requirements.

## Author Contributions

YO designed the study, managed data, undertook the statistical analysis, and wrote the first draft of the manuscript and figures. KY and YI managed the literature and revised the manuscript. All authors have read and approved the final manuscript.

## Conflict of Interest

The authors declare that the research was conducted in the absence of any commercial or financial relationships that could be construed as a potential conflict of interest.
